# The correct name for a subspecies of *Oenothera fruticosa* L. (Onagraceae)

**DOI:** 10.3897/phytokeys.34.7040

**Published:** 2014-01-31

**Authors:** Warren L. Wagner

**Affiliations:** 1 Department of Botany, MRC-166, National Museum of Natural History, Smithsonian Institution, P.O. Box 37012, Washington, DC 20013-7012, USA

**Keywords:** *Oenothera fruticosa*, *O. tetragona*, *O. glauca*, nomenclature

## Abstract

In 1978 when Straley adopted the name *Oenothera fruticosa* L. subsp. *glauca* (Michx.) Straley for one of the two recognized subspecies of *O. fruticosa* it was the correct name for this taxon; however, since that time the botanical code has changed so that now an autonym is treated as having priority over the name or names of the same date and rank that established it. This change means that since 1981 *O. fruticosa* subsp. *glauca* was no longer the correct name. The appropriate combination for it is made here as *O. fruticosa* L. subsp. *tetragona* (Roth) W.L. Wagner. Original material for the basionym, *O. tetragona*, is no longer extant so a neotype is designated.

## Introduction

The superfluous nature of the combination *Oenothera fruticosa* subsp. *glauca* (Michx.) Straley (1978) was brought to my attention by John Wiersema so that the nomenclature could be corrected in advance of the forthcoming treatment of *Oenothera* in the *Flora of North America*.

In a revision of *Oenothera* sect. *Kneiffia* (Straley 1978) a common eastern North American species widely cultivated in gardens, *Oenothera fruticosa*, was broadly delimited to include in synonymy a number of names previously recognized as distinct species. *Oenothera fruticosa* was subdivided into two widespread subspecies with subsp. *fruticosa* occurring at lower elevations and having a more southerly geographic distribution and subsp. *glauca* occurring in more northerly areas and at higher elevations. In 1978 Straley made the new combination *Oenothera fruticosa* subsp. *glauca* for the more northerly subspecies, based on *Oenothera glauca* Michx. and including among its synonyms *Oenothera tetragona* subsp. *glauca* (Roth) Munz and *Oenothera tetragona* Roth. In 1978 Straley’s new combination was the correct name for the taxon; however, since that time the botanical code of nomenclature has changed so that an autonym is treated as having priority over the name or names of the same date and rank that established it. This change means that since 1981, when this rule was added (Greuter 1981: 910, note 7), *Oenothera fruticosa* subsp. *glauca* was no longer the correct name for this taxon.

This rule is now Art. 11.6 of the current code of nomenclature (McNeill et al. 2012), and it dictates that the autonym, *Oenothera tetragona* subsp. *tetragona*, has priority over the name that created it, *Oenothera tetragona* subsp. *glauca*. Since the type of *Oenothera tetragona*, which is necessarily also the type of *Oenothera tetragona* subsp. *tetragona* (Art. 7.6), was definitely included by Straley in the synonymy of *Oenothera fruticosa* subsp. *glauca*, the correct name for this taxon must be the combination *Oenothera fruticosa* subsp. *tetragona*, which has not been previously made. The name *Oenothera fruticosa* subsp. *glauca*, although nomenclaturally superfluous (Art. 52.1), is not illegitimate (because it has a basionym; Art. 52.3) and is here placed into synonymy.

## Nomenclature

### Oenothera fruticosa tetragona

(Roth) W.L. Wagner comb. nov.

urn:lsid:ipni.org:names:77135763-1

http://species-id.net/wiki/Oenothera_fruticosa_tetragona

#### Basionym.

*Oenothera tetragona* Roth, Catal. Bot. 2: 39. 1800.

**Type.** Grown in the garden of Wilhelm Koch, at Gnadau, near Barby, Germany, of American origin; no extant material located. Pennsylvania. Lancaster Co.: between Churchtown Rd. and Beartown, 6 September 1892, A.A. Heller 549 (neotype, here designated: US-58278; isoneotypes: G-Bois, G, MO, NY). Munz (1937) apparently saw the original type material in B, stating “Ex horto meo Vegesackii, 1799, *Oe*, *tetragona*, Herb. A. W. Roth.” I contacted Robert Vogt at Berlin and there is currently no extant original material; however, he located a specimen grown from seeds provided by A. W. Roth “e semin. Rothianis in h. bot. Wratislav. colui 1823,“ but there is no way to clearly connect this material with the original material used when Roth described the species. I have here selected a neotype that fits Roth’s description and was considered to represent this entity in its narrowest interpretation. It was annotated by Munz for his 1937 revision and also by Straley for his 1978 revision.

#### Synonym.

*Oenothera glauca* Michx., Fl. Bor.-Amer. (Michaux) 1: 224. 1803. *Kneiffia glauca* (Michx.) Spach, Hist. Nat. Veg. Phan. 4: 374. 1835. *Oenothera fruticosa* L. var. *glauca* (Michx.) H. Lév., Monogr. Onothera 107. 1902. *Oenothera tetragona* Roth subsp. *glauca* (Michx.) Munz, N. Amer. Fl., ser. 2, 5: 91. 1965. *Oenothera fruticosa* L. subsp. *glauca* (Michx.) Straley, Ann. Missouri Bot. Gard. 64: 403. 1978 [“1977”; published 26 May 1978].

**Type.** Hab. in sylvis remotis et occidentalibus flumini *Mississippi* confinibus, versus regionem Illinoensium, 1787 or 1789, A. Michaux s.n. (holotype: P, photograph GH; isotype: P). According to Straley (1978) the type is labeled “Ouest de Ohio, Route aux Illinois,” but the plant is presumably from the southern Appalachians, in the Carolinas or Virginia where Michaux collected in June and July of 1787 and 1789.

## Supplementary Material

XML Treatment for Oenothera fruticosa tetragona
